# Supplementary material for: Noncanonical DNA Cleavage by BamHI Endonuclease in Laterally Confined DNA Monolayers Is a Step Function of DNA Density and Sequence
Source: Molecules. 2022 Aug 17;27(16):5262. doi: 10.3390/molecules27165262 (PMC9416302; doi:10.3390/molecules27165262)
Supplement: Supplementary file 1 [file molecules-27-05262-s001.zip › molecules-1819237-supplementary.pdf]

Supplementary Information

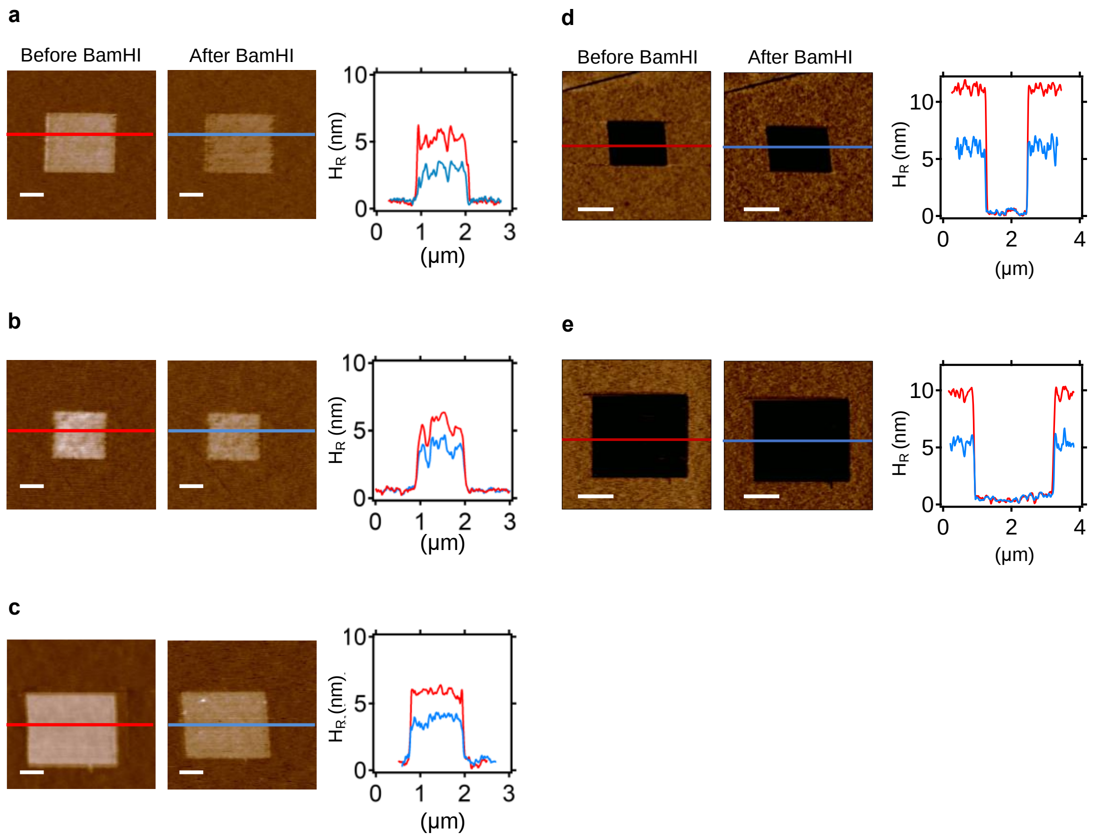

Figure S1. 50 nm width topographic profiles relative to AFM micrographs in Figs. 1-2, showing Noncanonical BamHI action respectively in LCDMs at intermediate DNA densities. Nanografted LCDMs in (**a**) (**b**) and (**c**) are those shown in top (DNA-1), middle (DNA-2) and bottom row (DNA-3) in Fig. 1, respectively. Negatively nanografted LCDMs in (**d**) and (**e**) are those shown in top (DNA-1) and bottom row (DNA-3) in Fig. 2, respectively. The scale bar (white) shown in the AFM micrographs is 500 nm in a-c, and 750 nm d-e, respectively.

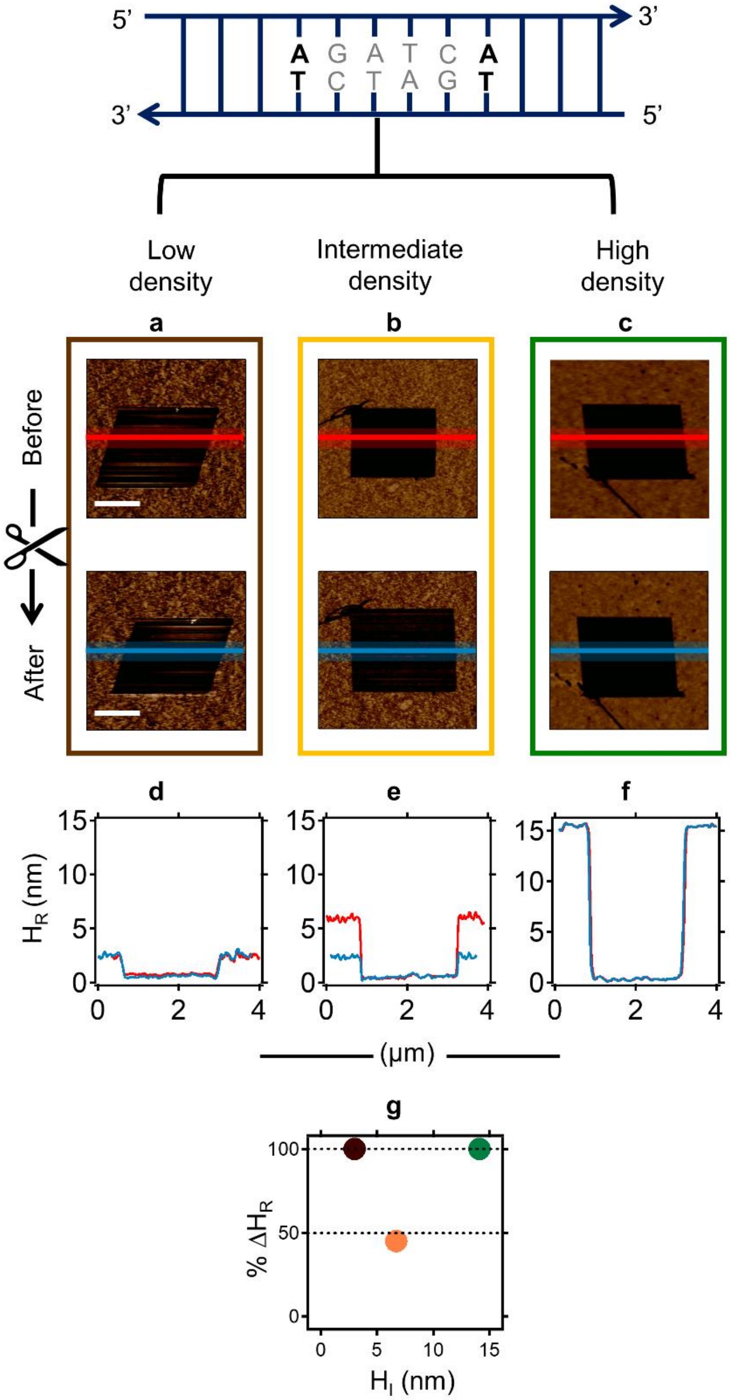

Figure S2. Sequence-density related activity of BamHI within self-assembled monolayers of DNA-3 in double-stranded(ds) form. The upper section shows the schematic representation of dsDNA-3 molecules with the noncanonical restriction site located in the middle. The core sequence of the site is written in grey while the two flanking bp are in bold black.. The density range is partitioned into three distinct subtypes: low density, intermediate density, and

high density (a-c, respectively). In frames (a-c) are displayed AFM micrographs of the confined DNA-3 molecules before (up) and after (down) BamHI action. The colour code (brown, orange, and green) designates low density, intermediate density and high density, respectively. Panels d-e represent AFM average line profiles, measured relative to the surrounding alkylthiol monolayer, across the AFM micrographs in the low-density frame, intermediate density frame, and the high-density frame. Panel (g) shows the relationship between the percentage change in the relative height (ΔH_R) of nanostructures in (a-c), as calculated from (d-e) against the initial height (H_I) of the LCDMs before the enzymatic reaction. The scale bar in (a) (white) is 1 µm and is the same for all the AFM micrographs in the Fig. The enzymatic reaction leads to no height reduction in the low-density regime, 50% reduction in relative height in the intermediate density regime, and no reduction in the relative height at the high-density regime. This results for DNA-3 SAMs shows a step-like activity of BamHI, determined by density and confinement, which is similar to our findings for DNA-2 nanografted patches (see Figure-3, middle column).

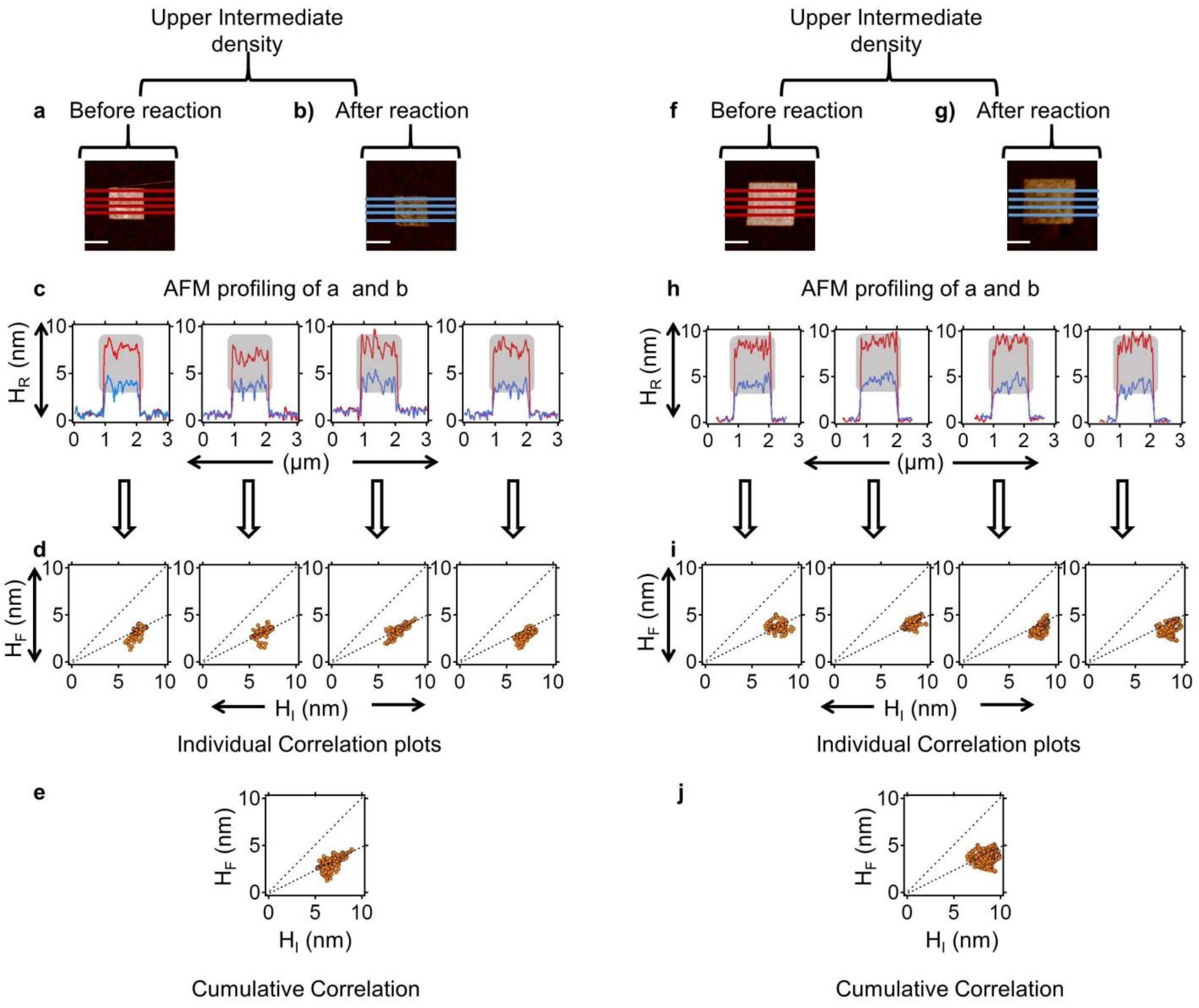

Figure S3. Catalytic action of BamHI within DNA-1 (left) and DNA-3 (right) LCDMs at upper intermediate densities. The first column (a-b) shows the two AFM micrographs that correspond to the DNA-1 LCDM before and after the BamHI action. Each LCDM was divided into four sectors, with line profile across each sector, and each line mapped to each another. The same procedure of data analysis was also performed on DNA-3 LCDM, as shown in (f, g). From the line profiles, individual height profiles were generated: four height profiles for DNA-1 LCDM in (c) and four for the DNA-3 LCDM in (h). Each height profile corresponds to the one-to-one mapping (red to light blue of the same subunit) of the line profile across (a, b) and (f, g), respectively. In all the height profiles in (c) and (h), the relative final height (light blue) of the LCDM is half of the relative initial height (red). This is shown in the individual correlation plots in (d) and (i), as all the height dots fall on the dashed line of 50% cleavage. Panels (e, j) display the cumulative correlation plot of all the individual plots in (d) and (i) respectively. The scale bar in a, b, f, g (white) corresponds to 750 nm.
